# Consequences of Inadequate Intakes of Vitamin A, Vitamin B_12_, Vitamin D, Calcium, Iron, and Folate in Older Persons

**DOI:** 10.1007/s13670-018-0241-5

**Published:** 2018-04-17

**Authors:** Jessica Watson, Marissa Lee, Maria Nieves Garcia-Casal

**Affiliations:** 1 Morsani College of Medicine, University of South Florida, Tampa, Florida USA; 2 Loma Linda University, School of Public Health, Loma Linda, CA USA; 3 Evidence and Programme Guidance Unit, Department of Nutrition for Health and Development, World Health Organization, 20 Av Appia, 1211 Geneva, Switzerland

**Keywords:** Vitamin A, Vitamin B_12_, Iron, Calcium, Vitamin D, Folate, Aging, Micronutrients, Older persons, Deficiency, Excess

## Abstract

**Purpose of Review:**

This review broadly discusses the consequences of inadequate consumption, by deficit or excess, of selected micronutrients on the quality of life and morbidity during aging, specifically considering increases in life expectancy and the costs of care in the older persons.

**Recent Findings:**

A literature review of the periods 2012 to 2018, focusing on vitamins A, B_12_, and D, calcium, iron and folate, was completed as these micronutrients are found to significantly affect the aging process. Causation and application of these micronutrients to disorders related to aging are controversial and mixed. This review highlights research needs and controversial points on the role of these micronutrients.

**Summary:**

Micronutrient deficiencies are a common and avoidable contributor to decreased quality of life and healthcare costs in the older persons. Further research is needed to determine adequate intakes and innovative uses, including appropriate thresholds for improved health outcomes for this population.

## Introduction

### Purpose

The United Nations *World Population Prospects* projects the number of persons aged 80 years + will triple from 137 million in 2017 to 425 million in 2050 [1]. The prevalence of chronic diseases, such as cancer, cardiovascular, and neurodegenerative diseases, is anticipated to rise along with this elderly population [2].

Nutrition contributes to overall health and improves treatment response for chronic diseases (i.e., immune response, mobility, cognitive function, healing) [2, 3]. A compromised health status can increase hospital length of stay and promote the development of comorbidities, contributing to additional costs of $13,350–$19,530 per hospitalization and $77,000 per year of care associated with poor nutrition status [4].

Aging is associated with an overall decline in physiological functions that contribute to inadequate nutritional status (i.e., decreased metabolic rate and gastric secretions, decreased sensory functions in oral cavity, increased use of medications, changes in overall body mass and electrolyte distribution) [2–5]. The cumulative effects of aging and micronutrient deficiencies in the older persons result in frailty, cognitive decline, poor immune response, cardiovascular disease, cancer, and other factors of morbidity [2, 6, 7]. On the other hand, the widespread use of dietary supplements (52% of adults in the USA report the use of at least one supplement product) has been reported to be of no benefit for some nutrients and could have deleterious consequences in this age group [8–11].

The purpose of this review is to update knowledge and briefly discuss the role and new findings of vitamins A, B_12_, and D, calcium, iron, and folate on morbidity and quality of life during aging, especially considering the increased life expectancy and the costs of care of this age group.

### Approach

A literature review of the periods 2012 to 2018 in older people aged 65+ years, focusing on vitamin A, vitamin B_12_, vitamin D, calcium, iron, and folate, was performed in MEDLINE and Google Scholar as these micronutrients are found to significantly affect the aging process [9–11]. Although the United Nations agreed cutoff is 60+ years to refer to the older population [12], developed world countries accept the age of 65+ years as the working definition of elderly [13]. Because most published studies have been done in developed nations, 65+ years of age was used for this review. The lists of references from selected articles were also searched.

The review focused on the consequences of inappropriate consumption of some vitamins and minerals—by excess or deficit—on the aging process itself and on the health and wellbeing of this age group, highlighting interactions between nutrients, research needs, and controversial points, when applicable. The risk of excessive intakes of vitamins and minerals is a topic that is getting more and more relevance in recent studies, due to the consequences for health on all age groups. For each nutrient, a brief description on the function and role in aging, the recommended nutrient intakes (RNI) [14], and the tolerable upper intake levels (UL) [15] were included (Table 1).

**Table 1:** Recommended nutrient intakes (RNI) and tolerable upper intake levels (UL) for selected micronutrients in the elderly (65+ years)

Nutrient	Recommended nutrient intake (RNI)^1^	Tolerable upper intake levels (UL)^2^
Vitamin A	600 μg/day	3000 μg/day
Vitamin B_12_	2.4 μg/day	ND
Folate	400 μg/day	1000 μg/day
Vitamin D	15 μg/day	50 μg/day
Calcium	1300 mg/day	2500 mg/day
Iron	Based on diet availability (15, 12, 10, 5%)	
Male	9, 11, 14, 27 mg/day	45 mg/day
Female	8, 9, 11, 23 mg/day	45 mg/day

Sources: WHO/FAO, 2005 [14]) ^1^ (IOM, 2000 [15]) ^2^

## Findings

### Vitamin A

#### Importance of Vitamin A in Aging

Vitamin A has several important roles in the aging process: mainly immune function and oxidative processes. Deficiency is associated with defective immune response to infection. All-trans retinoic acid (RA), a common form of active vitamin A, plays a role in immune homeostasis through regulating cell homing and differentiation. During times of infection or autoimmune disease, it activates T cell responses [16]. Emerging research is focusing on the effects of vitamin A enhancing T cell responses to cancer, infection, intestinal inflammation, and immune-mediated diseases in humans including autoimmune disease—all associated with aging [16].

##### Deficiency

Effects of vitamin A deficiency include neurodegeneration, physiological function of steroid and thyroid hormones, and historically, vision and skin changes. All-trans RA has protective effects in neurodegeneration**.** It diminishes the production of amyloid-β peptides and their oligomerizations in the development of Alzheimer’s disease [17].

i. Vision
  Vitamin A roles on vision and skin changes are well studied. The carotenoids lutein and zeaxanthin confer the most benefit to the retina in age-related macular degeneration. However, vitamin A and other carotenoids are inconsistently associated with prevention or improvement in macular degeneration [18].

ii. Skin
  Age-related complications and diseases are associated with decreased retinoid signaling and the decline of various hormones, including steroids. RA plays a role in activating RARs and RXRs, steroid thyroid hormone receptor transcription factors. In 2015, it was reported that RA may restore steroidogenesis in epidermal keratinocytes of aged individuals [19]. Additionally, retinol increases epidermal thickness by upregulation of transcription factors, collagen genes responsible for wound healing, improving extracellular matrix microenvironment, extracellular matrix production, and activating dermal fibroblasts [20, 21].

##### Excess

A recent review reports that only a small percent of the older persons have intakes below the RNI, showing that in fact vitamin A intake in this age group is usually much higher than the RNI [22•]. Vitamin A excess is of special concern in the older persons who may have difficultly clearing it [22•]. Chronic vitamin A excess is associated with loss of hair, dry mucous membranes and skin, cortical bone loss and fractures, and potentially an increased risk of mortality [23, 24]. Vitamin A is a clear example of the importance of maintaining appropriate micronutrient intakes and concentrations avoiding extremes, both high and low.

i. Bone Fractures
  Reported health outcomes associated with excess vitamin A include premature skin aging in elderly HIV patients and increased risk of bone fractures [25, 26]. Retinol and carotenoids may support a chemo-preventive phytochemical role in estrogen-dependent cancers, common in post-menopausal women. β-Carotene levels were independently and inversely associated with estradiol levels in older women [27]. Previous reports on associations between vitamin A intake, serum retinol concentrations, and bone mineral density or fracture are controversial. The inconsistencies may be due, in part, to difficulties in obtaining an accurate assessment of vitamin A since it has been reported that serum retinol is a poor proxy for vitamin A status [28, 29].

#### Interaction Between Nutrients

Vitamin A, a fat-soluble vitamin, is absorbed with vitamin D. Thus, serum retinol should always be assessed in the presence of vitamin D supplementation [23]. New research has found that retinoic acid and vitamin D calcitriol combined influence proliferation and differentiation of osteoblasts [30]. An increase in PPARγ2 expression was observed following retinoic acid administration, whereas a decrease was observed following calcitriol administration [30]. Additionally, cumulative serum retinol and zinc levels among older persons may help in high-frequency hearing loss [31]. Similarly, zinc supplementation in individuals aged 70–85 years increased vitamin A levels proportionately with zinc [32].

#### Gaps in Research—Further Research

Leukocyte telomere length (LTL) is a biomarker of the aging process. These repetitive sequences of DNA at the ends of chromosomes serve to protect the ends of DNA strands and are associated with risk of chronic disease. High oxidative stress can lead to DNA strand breaks, shortening telomeres. Several serum carotenoids, including β-carotene, β-cryptoxanthin, lutein, and zeaxanthin), were found to be associated with LTL. Vitamin A roles in immune function, inflammation, regulation of gene expression, and epigenetic modification, help maintain LTL [33•]. More research is needed to understand the association between vitamin A, LTL, and the potential use of this association during the aging process.

The potential for exacerbating an already serious public health problem, with intakes of vitamin A currently considered safe, indicates further research into this matter is needed.

### Vitamin B_12_ and Folate

#### Importance of Vitamin B_12_ and Folate in Aging

Vitamin B_12_ and folate are associated with preventing chronic diseases associated with aging through the methylation of homocysteine [34]. This is a vital process preventing amyloid and tau protein accumulation which can cause cognitive decline [34]. The genetic polymorphism in the MTHFR 677TT gene is hypothesized to be a main contributor to metabolism in aging processes [35•]. Elevated homocysteine and lower vitamin B levels impact the immune system, causing increased inflammation and antioxidant damage which catalyze physiological aging in all systems [35•].

##### Deficiency

Vitamin B_12_ deficiency can lead to megaloblastic anemia and demyelinating neurological symptoms, including irreversible nerve damage and neuropathy. Older adults are at high risk of vitamin B_12_ malabsorption due to the lack of production of intrinsic factor [36]. Unfortunately, symptoms are often misdiagnosed because they mimic Alzheimer’s disease and other chronic conditions with non-specific symptoms [2]. Folate deficiency is associated with a poor diet and malabsorptive disorders. It can result in megaloblastic anemia, soreness, and shallow ulcerations in tongue and oral mucosa, and changes in hair, skin, and fingernails [37•].

i. Cognitive Decline
  Vitamin B_12_ and folate have a strong protective effect in mental health decline in the older persons [38]. Decreased dietary vitamin B_12_, folate, and vitamin B_6_ was shown to precede depression in this age group; while vitamin B_12_ was found to have protective effects against depression [39, 40].
  Folate and vitamin B_12_ deficiencies result in increased total homocysteine levels associated with faster rates of total brain volume loss and cause severe neurocognitive decline [41, 42]. One study found high vitamin B_12_ levels were associated with a decreased rate of total brain volume loss, while folate levels were not [43]. On the other hand, other studies show that vitamin B_12_ was not associated with dementia and cognitive decline compared to folate. A large longitudinal study of elderly patients at risk of dementia found that higher dietary folate intake was associated with a decreased risk of dementia [44]. Another study found folate supplementation of 800 μg/day positively affected cognitive decline through significantly improving memory, sensorimotor speed, and information processing [34]. A study in 2016 reported that increases in folate intake in older persons worsened clinical outcomes in peripheral neuropathy associated with vitamin B_12_ deficiency [36].

ii. Cardiovascular Disease
  In older persons, high serum homocysteine and folate along with low vitamin B_12_ concentrations have been reported to increase the risk of cardiovascular disease (CVD) showing that there was strong evidence connecting stroke and heart disease with folic acid levels, treatment, and supplementation [35•]. Low vitamin B_12_ levels were found to increase blood pressure, contributing to CVD. In addition, decreases in vitamin B_12_ concentrations can result in hyperhomocysteinemia, which could quadruplicate elderly patients’ risk of stroke due to atrial fibrillation [45].

iii. Bone Health
  Folate and vitamin B_12_ have also been associated with bone health. Interestingly, elevated levels of homocysteine have been associated with fracture risk and bone mineral density (BMD) [35•]. Studies have found the MTHFR 677 polymorphism to be associated with a 23% increased risk of all fractures through changes in BMD. However, intervention studies using folate and vitamin B_12_ have reported no significant associations, suggesting that B vitamin interventions may be confined to at-risk groups with sub-optimal vitamin B_12_ and folate levels [35•].

##### Excess

There are no known adverse effects of large intakes of vitamin B_12_; rather, deficiency is of utmost concern [46]. A more pressing problem in regard to the older persons is excessive folate intake. In the USA, those 50 years and older have the highest folate intake and may be at risk of excessive folate intake due to supplementation and fortified foods [47]. Higher levels of folate have historically been seen to worsen anemia and cognition while masking low levels of vitamin B_12_. Another major concern in excess folate is its controversial and complex dual role in colorectal cancer. While some studies have found folate to be protective in risk of colorectal cancer, other studies have found folate supplementation to be potentially cancer-promoting [48]. Thus, the optimal intake for those over 65 years is unknown.

#### Interaction Between Nutrients

As the older persons have increased consumption of gastric acid inhibitors, they are at increased risk of vitamin B_12_ deficiency due to the lack of vitamin B_12_ absorption. Calcium supplementation has been found to positively affect the association between gastric acid inhibitors and vitamin B_12_ deficiency [49].

#### Gaps in Research—Further Research

It is still unknown to what extent B vitamins involved in one-carbon metabolism can affect DNA methylation throughout the life cycle. Some studies have found that long-term supplementation with folic acid and vitamin B_12_ in elderly subjects resulted in effects on DNA methylation of several genes, including those implicated in developmental processes. Furthermore, more research is needed on the effect of both folate and B_12_ on cognitive function, CVD, and bone health as they relate to aging. Public health conclusions in regard to folate and vitamin B_12_ point to fortification of foods, but safe upper level intake of folic acid and its interaction with vitamin B_12_ needs to be further researched. This is especially urgent in regard to the potentially positive role excess folate contributes to promoting colorectal cancer.

### Iron

#### Importance in Aging

Iron is an essential functional component of vital metabolic functions such as oxygen transport, oxidative energy production, inactivation of harmful oxygen radicals, and DNA synthesis. It has a nutrigenomic effect in the central nervous system as a cofactor for oxidative phosphorylation, neurotransmitter production, nitric oxide metabolism, and oxygen transport, playing an important role in neuroprotection and neuronal activities [50].

Studies reveal iron is essential to maintain immune and antioxidant function during aging [51]. Iron status becomes impaired in situations that involve chronic inflammation, such as obesity or aging [50, 52] and malnutrition exacerbates these effects of inflammation [53•]. Inflammation affects hepcidin, a peptide hormone that regulates iron homeostasis [53•].

Anemia and iron deficiency are two of the most prevalent disorders worldwide, affecting people in all regions and ages, including older persons. Iron overload, although usually associated to pathological conditions, also has important consequences on health and could be of relevance during aging [54].

##### Deficiency

Post-menopausal women and older men are at an increased risk of iron deficiency because of chronic blood loss from disease conditions, decreased absorption due to reduced acid secretion, use of medications such as antacids, or a diet low in iron [2, 48]. The current RNI and UL for iron (Table 1) are controversial because of the effect that age, gender, race, and ethnicity could have on the level and functionality of iron [52].

i. Anemia and Depression

The most common form of nutritional anemia, iron deficiency anemia, is associated with impaired cognitive performance, depressive symptoms, reduced quality of life, and increased hospitalization and mortality in the older persons [53•, 55, 56]. According to the World Health Organization definition of anemia, over 10% of community-dwelling adult aged ≥ 65 are classified as anemic [57, 58]. The prevalence of anemia increases significantly with advancing age and exceeds 20% in those aged 85+ years [55]. A NHANES study from 2004 found that out of those diagnosed with anemia, only one third had nutritional deficiency, primarily of iron [59].

Depressive symptoms were significantly higher in a cross-sectional study of 1875 elderly men and women across England with low iron status (low Hb and high sTfR) [60]. However, chronic illness decreased the association and depressive symptoms were not associated with ferritin, suggesting anemia of chronic disease and not necessarily an iron intake problem [53•].

##### Excess

Iron-overloaded states, especially hemochromatosis, a disease resulting in excess levels of iron in the liver, results in increased iron-induced cell damage, fibrosis, cirrhosis, and hepatocellular cancer [54]. Those with iron overload are also at increased risk of infection. Pathogens also benefit for administered iron, causing caution with iron supplementation, which promotes health in iron deficiency, but require previous treatment of any ongoing infection [61].

i. Cardiovascular Disease
  Increased tissue iron stores have been associated with increased risk of a number of chronic diseases, including heart disease, diabetes mellitus, and potentially cancer in middle-aged and older populations [53•]. Positive associations have been found between ferritin, low serum iron, and cardiovascular disease markers in elderly population [53•, 62, 63]. However, causality could not be indicated because of similar concerns of biomarkers used and the discrepancies between inflammation and iron serum levels.
ii. Neurodegenerative Disorders
  During aging, iron accumulates in different brain regions associated with motor and cognitive impairments. The link between inflammation and iron is complex, but inflammatory mediators (such as tumor necrosis factor alpha, interleukin-6, and ferroportin-1) stimulate hepcidin, thereby upregulating iron deposition in the brain. This disruption in homeostasis has been seen on magnetic resonance imaging, a potential diagnostic biomarker of neurodegenerative disorders [64], although there has not been clear proof of a causal relationship. The associated oxidative damage from brain iron accumulation could be responsible for pathogenesis of dementia [61]. More studies are needed to see if iron-induced oxidation contributes to Alzheimer’s disease and Parkinson’s disease.

iii. Iron-Overload, Cancer, and Infection
  Because iron interacts with DNA at a genomic level, high iron levels could be involved with specific cancer growth and angiogenesis [65, 66]. Iron is pro-inflammatory and promotes oxidative damage. Its carcinogenicity has been initially researched in animal studies [67]. However, a broad review by the Scientific Advisory Committee on Nutrition found insufficient data regarding a true link between iron and colorectal cancer [68].

#### Interaction Between Nutrients

Vitamin C might increase the absorption of dietary iron by up to 10% and calcium decreases iron absorption. Therefore, if both are needed, it is advised to take iron and calcium hours apart [69, 70]. Additionally, if taken without food, large doses of iron supplements impair the absorption of zinc [50].

#### Gaps in Research—Further Research

The diagnosis of iron deficiency anemia is highly controversial. Based on the differences in iron absorption and chronic inflammation, as well as the prevalence of comorbidities, the diagnosis of iron deficiency and iron excess in the older persons needs specific review. It may also be appropriate for future diagnosis standards of iron deficiency to be based on differences in iron absorption, age, gender, and race.

### Vitamin D and Calcium

#### Importance of Vitamin in Aging

Vitamin D intake and absorption drastically decreases with age due to decreased sunlight exposure, nutrient intake, fat absorption, and conversion of vitamin D to its active form [2]. In regard to aging, vitamin D is associated with cognition, depression, cancer, and cardiovascular disease [10, 24, 71]. Vitamin D stimulates clearance and phagocytosis by macrophages, protecting immune cells against apoptosis by regulating both extranuclear protein functions and gene expression signaling [72].

Calcium and vitamin D interact to effect vasculature, lipid metabolism, and neuromodulation. Strongly influenced by age, the efficiency of calcium absorption from the gastrointestinal tract decreases significantly after age 60 in both sexes: with those aged 70 years and older absorbing approximately one-third less calcium than do younger adults. Women are at an even greater risk of decreased calcium because of a decreased fractional calcium absorption after menopause, estrogens acceleration on bone loss, and increased urinary calcium losses [73, 74].

##### Deficiencies

In 2013, 40–100% of elder populations globally were below the WHO standard of vitamin D sufficiency (< 50 nmol/L) [11, 75, 76]. Yet, some groups have found changes in cognition at significantly lower thresholds (< 10 ng/mL) [72]. Inadequate vitamin D and calcium has been linked with many conditions and symptoms. In a recent systematic review of micronutrient intakes in older persons, nearly 65% of the population of men and 73% of the women studied were at risk of inadequate intakes of calcium based on the RNIs in Table 1 [77•].

i. Falls and Fracture Risk
  Fracture risk, bone loss, and the resulting osteoporotic fractures are the predominant bone health concern for those over 70 years of age. Vitamin D and calcium, alongside of exercise, are well-known preventive measures for preventing bone loss in the older persons [73, 78].
  Current data revels the beneficial effect of vitamin D supplementation on muscle strength, physical performance, and prevention of falls and fractures in elderly female populations [79]. While lower 25-[OH]D levels in institutionalized older persons were associated with hip fractures, falls alone were hardly associated to vitamin D status [80]. Interestingly, elevated parathyroid hormone (PTH) was inversely correlated with vitamin D status and was the only predictor of hip fractures [81]. Additionally, accumulating evidence suggests vitamin D deficiency is associated with sarcopenia in elderly women [82].
  Overall, there is good evidence (a review of 17 RCTs) that vitamin D (doses < 800 IU) plus calcium (500 mg) results in small increases in bone mineral density (of the spine, total body, femoral neck, and total hip). However, it is less certain if vitamin D alone improves bone density. Calcium supplementation alone was not significantly different than the combination of vitamin D plus calcium, suggesting that calcium alone may help replete bone mineral density [83•].
ii. Cognition
  The existing body of evidence provides proof that people with 25-[OH]D concentrations < 10 ng/mL have the greater risk of cognitive disorders [84]. However, vitamin D’s role in cognitive decline remains unclear.
  Previously, low vitamin D concentrations have been associated with worse cognitive performance and cognitive decline than persons with adequate levels. However, several vitamin D supplementation studies in large elderly cohorts showed no significant improvement in cognition compared with controls [85–87]. There is a need for more studies on true association of vitamin D and calcium on cognition in elderly adults, a clear definition of cognition, and appropriate thresholds for vitamin impacts on cognition.

##### Excess

Vitamin D excess is measured through hypercalciuria and hypercalcemia (25(OH)D) levels above 88 ng/mL). Serious consequences of vitamin D overuse are acute kidney injury and pancreatitis, secondary to hypercalcemia. The increase in supplementation has been a cause for concern due to the inconsistencies in safe vitamin level intake [11, 73, 88]. The thresholds of vitamin D are widely debated [77•]. In 2010, more than 50% of older men and almost 70% of older women in the USA were taking supplemental calcium [89]. Non-skeletal effects of calcium, especially potential adverse effects on cardiovascular health, are not well established and remain controversial. Over-supplementation still needs to be studied for both of these vitamins as adverse effects are unknown and current supplementation to prevent poor health outcomes.

i. Cardiovascular Disease and Mortality
  Following inconclusive results of vitamin D reduction of cardiovascular disease and all-cause mortality, a recent review of vitamin D found that higher levels of vitamin D had inverse association with mortality probably through an effect of vitamin D on lipid status [71, 83•]. A study on excess dietary calcium intake was associated with higher Framingham Risk Score (FRS), a proxy measure of cardiovascular health. This study suggests that excess (> 1200 mg/day) as well as very low (< 300 mg/day) dietary calcium intakes is related to higher FRS in both genders [90]. However, two comprehensive reviews including over 87 clinical trials, 100 literature reviews, and 200 primary articles could not support claims on the combined effect of vitamin D and calcium on cardiovascular health or all-cause mortality [83•, 91•].

#### Interactions Between Nutrients

Vitamin D forms the basis of calcium’s homeostatic mechanism. Calcium and vitamin D interact through regulated feedback and are dependent on each other for maintenance of appropriate levels. Vitamin D insufficiency may result in a relative hypocalcemia and high serum PTH concentrations, which alone has been linked to poor health outcomes [73]. This secondary hyperparathyroidism can be attenuated by the administration of vitamin D supplements. Calcium can compete or interfere with the absorption of iron, zinc, and magnesium [92] and must be addressed if older persons are at risk of these deficiencies.

#### Gaps in Research—Further Research

Due to vitamin D’s unique activation processes (requiring sun exposure), accurate levels of RNI are hard to discern and are widely debated [73]. Those that are institutionalized, homebound, have limited sun exposure, dark skin, or malabsorption are at increased risk for vitamin D deficiency and recommended to consume more with a goal of achieving levels of ≥ 30 ng/mL [93]. A cohesive definition of vitamin D insufficiency must be addressed. Vitamin D thresholds for deficiency and excess need to be reviewed for all age groups, since the signs and symptoms of deficiency are not always related to the current cutoff points.

Most research relating calcium and aging has been in conjunction with vitamin D. Yet, according to recent findings, calcium may be a critical nutrient in neurocognitive protection and bone health. Recent reviews have not found a clear link between many of the recommended intake levels of vitamin D or calcium with improvements in health outcomes and more research is needed to determine accurate thresholds for the elderly. Additionally, more robust research is needed to determine causation of vitamin D on health outcomes and dose-dependent effects of both vitamin D and calcium on bone health [9, 71, 82].

## Limitations/Implications

This review did not include all micronutrients contributing to the aging process and health outcomes in the elderly (i.e., thiamin, riboflavin, magnesium, selenium, zinc, or vitamin C) which will be important for further reviews as current evidence reveals inadequate intakes in the elderly population and consequential harm [77•].

Overall, there are insufficient studies on the elderly age group and on different outcomes by gender. Within the older persons, studies must sub-classify and subgroup cohorts. For example, a recent systematic review on all health outcomes associated with vitamin D contained very limited information on individuals older than 70 years. It would be interesting to consider subgrouping ages in the old people group, especially for some nutrients and functions that are not necessarily the same for 70- or 80-year-old individuals.

Due to the brevity of this review, details and limitations of each review presented were not mentioned. Populations studied, sample size, and overlapping age ranges may not be generalizable to all elderly. In addition, the detail we were able to provide on each study was limited.

## Final Comments

As the use of supplements increases and prevention is emphasized, the application of these micronutrients should be adequately studied for safe treatment recommendations in the elderly population. Low dietary intakes and all micronutrient deficiencies mentioned in this review are associated with functional decline, decreased quality of life, and increased healthcare costs. However, over supplementation is also dangerous and more studies on the subclinical effects and interactions with other nutrients are needed.

Seeking to understand the definitive thresholds of the different vitamins and minerals on their association with health outcomes, both in deficiencies (such as vitamin D) and excess (such as vitamin A and calcium), is important for setting clinical guidelines and policies. There is a need for more robust and focused research to determine micronutrient effects and application to medical conditions related to aging, such as neurocognitive disorders, cardiovascular disease, and cancer.

Finally, it is difficult to assess the value of nutrition interventions since most clinical trials do not assess the costs acquired from morbidity and mortality secondary to malnutrition. More studies are needed to determine the link of nutritional deficiencies with hospital length of stay, preventable healthcare use, and costs.

## Conclusion

A literature review of the periods 2012 to 2017, focusing on vitamin A, vitamin B_12_, vitamin D, calcium, iron, and folate, was completed and these micronutrients are found to significantly affect the aging process. Micronutrient inadequacies are a common and avoidable contributor to decreased quality of life in the elderly. Further research is needed to determine adequate intakes, including appropriate thresholds for health outcomes and safety from over supplementation for this population.
